# Biochemical characterization of three new α-olefin-producing P450 fatty acid decarboxylases with a halophilic property

**DOI:** 10.1186/s13068-019-1419-6

**Published:** 2019-04-08

**Authors:** Yuanyuan Jiang, Zhong Li, Cong Wang, Yongjin J. Zhou, Huifang Xu, Shengying Li

**Affiliations:** 1 Shandong Provincial Key Laboratory of Synthetic Biology, CAS Key Laboratory of Biofuels, Qingdao Institute of Bioenergy and Bioprocess Technology, Chinese Academy of Sciences, No. 189 Songling Road, Qingdao, 266101 Shandong China; 2 University of Chinese Academy of Sciences, Beijing, 100049 China; 3 Division of Biotechnology, Dalian Institute of Chemical Physics, Chinese Academy of Sciences, Dalian, 116023 China; 4 State Key Laboratory of Microbial Technology, Shandong University, Qingdao, 266237 Shandong China; 5 Laboratory for Marine Biology and Biotechnology, Qingdao National Laboratory for Marine Science and Technology, Qingdao, 266237 Shandong China

**Keywords:** P450 fatty acid decarboxylase, Fatty acid, Alkene, Biofuel, Halophilic enzymes

## Abstract

**Background:**

The CYP152 family member OleT_JE_ from *Jeotgalicoccus* sp. ATCC 8456 has been well-known to catalyze the unusual one-step decarboxylation of free fatty acids towards the formation of terminal alkenes. Efforts to tune up its decarboxylation activity for better production of biological alkenes have been extensively explored via approaches such as site-directed mutagenesis and electron source engineering, but with limited success. To gain more insights into the decarboxylation mechanism and reaction bifurcation (decarboxylation versus hydroxylation), we turned to an alternative approach to explore the natural CYP152 resources for a better variety of enzyme candidates.

**Results:**

We biochemically characterized three new P450 fatty acid decarboxylases including OleT_JH_, OleT_SQ_ and OleT_SA_, with respect to their substrate specificity, steady-state kinetics, and salt effects. These enzymes all act as an OleT_JE_-like fatty acid decarboxylase being able to decarboxylate a range of straight-chain saturated fatty acids (C_8_–C_20_) to various degrees. Site-directed mutagenesis analysis to the lower activity P450 enzyme OleT_SA_ revealed a number of key amino acid residues within the substrate-binding pocket (T47F, I177L, V319A and L405I) that are important for delicate substrate positioning of different chain-length fatty acids and thus the decarboxylation versus hydroxylation chemoselectivity, in particular for the mid-chain fatty acids (C_8_–C_12_). In addition, the three new decarboxylases exhibited optimal catalytic activity and stability at a salt concentration of 0.5 M, and were thus classified as moderate halophilic enzymes.

**Conclusion:**

The P450 fatty acid decarboxylases OleT_JE_, OleT_JH_, OleT_SQ_ and OleT_SA_ belong to a novel group of moderate halophilic P450 enzymes. OleT_JH_ from *Jeotgalicoccus halophilus* shows the decarboxylation activity, kinetic parameters, as well as salt tolerance and stability that are comparable to OleT_JE_. Site-directed mutagenesis of several key amino acid residues near substrate-binding pocket provides important guidance for further engineering of these P450 fatty acid decarboxylases that hold promising application potential for production of α-olefin biohydrocarbons.

**Electronic supplementary material:**

The online version of this article (10.1186/s13068-019-1419-6) contains supplementary material, which is available to authorized users.

## Background

Development of renewable, sustainable, and cost-effective biofuels has been driven by the shortage of fossil fuels, serious environmental problems, and ever-changing geopolitical factors [1, 2]. Among different types of biofuels, biohydrocarbons have attracted much attention as an ideal alternative to petroleum-based fuels due to their high energy content, low hygroscopicity, and compatibility with existing engine and distribution systems [3–7].

**Fig. 1:**
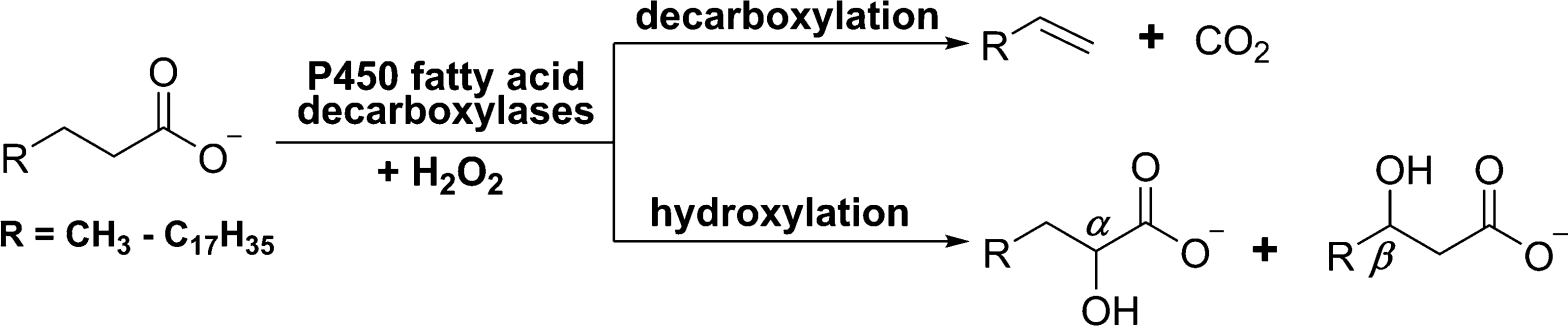
Decarboxylation and hydroxylation reactions catalyzed by P450 fatty acid decarboxylases

Cytochrome P450 (CYP) enzymes are a superfamily of proteins with a thiolate-heme prosthetic group, which are renowned for their versatile catalytic activities and exceptional capability to accept a vast variety of substrates [8–10]. According to the catalytic properties, P450 enzymes can be classified into monooxygenases, peroxidases, and peroxygenases [10]. The biofuel-related P450 fatty acid decarboxylases (FADCs), such as OleT_JE_ from *Jeotgalicoccus* sp. ATCC 8456 [11] and CYP-Sm46 from *Staphylococcus massiliensis S46* [12], belong to the CYP152 peroxygenase family. This family of P450 enzymes utilizes H_2_O_2_, instead of O_2_ which is employed by most of P450 monooxygenases, as the oxidant to support the unique oxidative decarboxylation reactions (Fig. 1) that convert the *C*_*n*_ (*n* = 4–22) chain length free fatty acids (FFAs) into *C*_*n*−1_ chain length 1-alkenes (i.e., α-olefins) [13, 14]. Since α-olefins are both excellent biofuel molecules and useful precursors of lubricants, detergents and other chemicals [15, 16], P450 FADCs hold promising application potential for production of biological α-olefins.

A majority of CYP152 peroxygenases catalyze FFA hydroxylation and decarboxylation reactions simultaneously. According to the main catalytic reaction types, this family of P450 enzymes can be classified into P450_BSβ_-like FFA hydroxylases (i.e., those with FFA hydroxylation as major chemistry, such as P450_BSβ_ mainly produces similar amounts of α- and β-hydroxyl fatty acids, P450_SPα_ and CYP-Aa162 mainly catalyze the C_α_ hydroxylation of FFAs, CYP-MP introduces the hydroxyl group at various carbon positions, and OleT_MC_ prefers to hydroxylate the long-chain fatty acids) and OleT_JE_-like FFA decarboxylases (i.e., those preferentially catalyze FFA decarboxylation reaction, like OleT_JE_ and CYP-Sm46Δ29) [11, 12, 14, 17–19]. To understand the unusual decarboxylation mechanism for activity and selectivity optimization, a growing number of studies on the prototypic P450 FADC OleT_JE_ have been carried out [20–29]. For example, Munro et al. resolved the crystal structures of wild-type OleT_JE_ [28] and a number of mutants such as R245L/E, F79A/Y/W and H85Q [30], revealing the important roles of Arg245, His85, and Phe79 in both catalytic activity and decarboxylation/hydroxylation bifurcation, as well as a group of active site residues responsible for productive fatty acid substrate binding. Furthermore, systematic mutagenesis analyses of a select group of active site residues including Arg245, His85 and Ile170 suggested the accurate substrate positioning is essential for decarboxylation activity [19, 27, 30, 31].

Interestingly, OleT_JE_, the first identified P450 FADC remains the best biocatalyst for α-olefin production in terms of both catalytic efficiency and chemoselectivity (i.e., decarboxylation versus hydroxylation) when compared to other biochemically characterized P450 FADCs including P450_BSβ_, CYP-MP, OleT_MC_, CYP-Aa162, and CYP-Sm46Δ29 [12, 19, 32]. Of note, some approaches such as redox partner engineering [31] and development of photocatalytic systems [33, 34] were also unsuccessful to improve the decarboxylation activity of OleT_JE_. Thus, it is highly expected to discover or engineer a novel CYP152 biocatalyst that can convert FFAs to 1-alkenes more efficiently and selectively.

In this work, we in vitro characterized three novel CYP152 FADCs including OleT_JH_ from *Jeotgalicoccus halophilus* (CYP152L1_ortholog, GenBank accession number: WP_092595307), OleT_SQ_ from *Salinicoccus qingdaonensis* (CYP152L8, WP_092983663), and OleT_SA_ from *Staphylococcus aureus* (CYP152L7, WP_049319149). We determined the substrate preference, kinetic parameters and salt tolerance of these enzymes for the first time. Moreover, site-directed mutagenesis on OleT_SA_, the OleT with relatively lower activity, was performed in order to further understand the function of active site residues in the catalytic activity and chemoselectivity of P450 FADCs.

## Results

### Genome mining of OleT_JE_-like P450 fatty acid decarboxylases

**Fig. 2:**
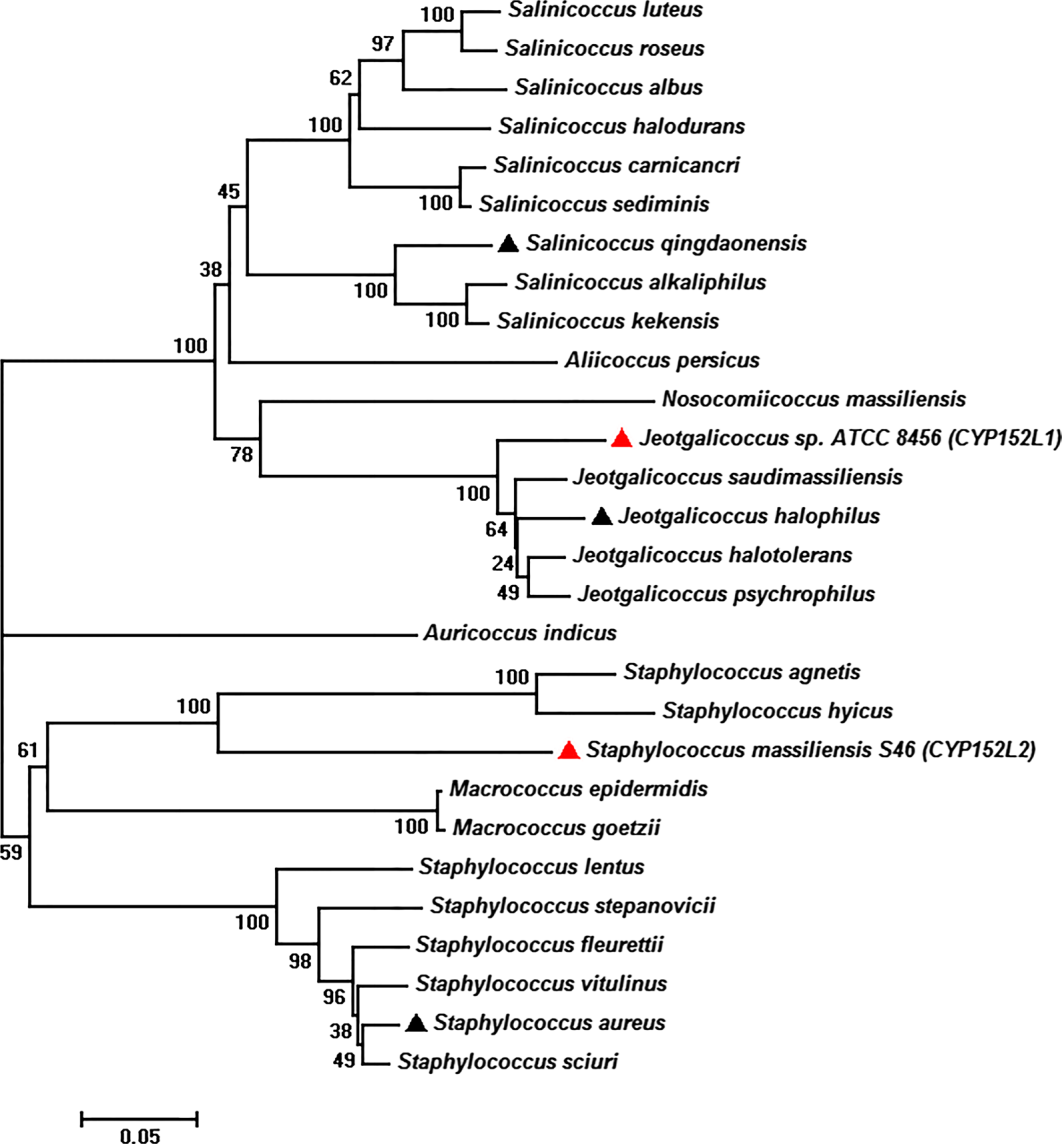
Phylogenetic tree analysis for CYP152 FADCs. The phylogenetic tree was built from the protein sequences of OleT_JE_ (CYP152L1), CYP-Sm46Δ29 (CYP152L2) and the select number of CYP152 family members with their protein sequence homology greater than 60%, using the neighbor-joining method. The strain names denote the source of the corresponding OleT enzymes. The two reference enzymes are marked by red triangles. The enzymes characterized in this study are marked by black triangles

The number of biochemically characterized P450 FADCs is far less than that of existing CYP152 sequences with potential FFA oxidation activities in GenBank. To explore more P450 FADCs that may possess greater decarboxylation activity and/or selectivity, we built a phylogenetic tree (Fig. 2) based on the protein sequences of the two known P450 FADCs OleT_JE_ (CYP152L1) and CYP-Sm46Δ29 (CYP152L2), and their homologous sequences with a sequence identity higher than 60%. Interestingly, these sequences mostly originate from the genera of *Jeotgalicoccus*, *Staphylococcus*, and *Salinicoccus*, which are well-known microorganisms associated with halophilicity or salt tolerance [35–38]. This finding suggests a possibility of identifying more 1-alkene producers and FADCs with higher decarboxylation activities from these groups of microorganisms.

Next, based on the conserved residues that are believed to be essential for the decarboxylation activity, including Phe79, His85, Ile170 and Arg245 (OleT_JE_ numbering, Additional file 1: Figure S1), we from each major branch selected OleT_JH_ from *Jeotgalicoccus halophilus* (CYP152L1_ortholog, GenBank accession number: WP_092595307), OleT_SQ_ from *Salinicoccus qingdaonensis* (CYP152L8, WP_092983663), and OleT_SA_ from *Staphylococcus aureus* (CYP152L7, WP_049319149) for our following biochemical characterization. According to the protein sequence alignment (Additional file 1: Table S1 and Additional file 2), OleT_JH_, OleT_SQ_, and OleT_SA_ show 93% (62%), 76% (63%), and 69% (64%) amino acid sequence identity to OleT_JE_ (CYP-Sm46Δ29), respectively.

### Substrate specificity and chemoselectivity

The codon-optimized genes that encode OleT_JH_, OleT_SQ_, and OleT_SA_ were individually expressed in *Escherichia coli* BL21(DE3). The resultant *N*-terminal His_6_-tagged recombinant proteins were purified to homogeneity using nickel affinity chromatography (Additional file 1: Figure S2). As expected, the three enzymes showed characteristic CO-bound reduced difference spectra (Additional file 1: Figure S3), indicative of their functional expression.

**Fig. 3:**
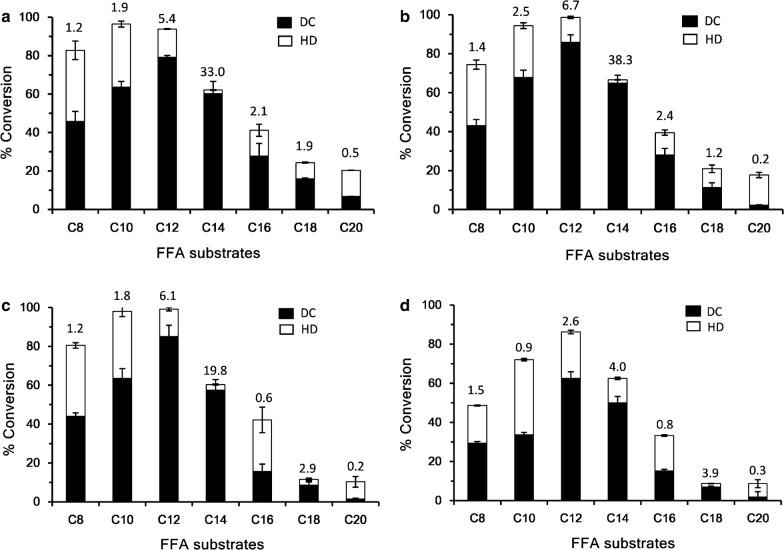
Substrate preference and product distribution profiles of OleT_JE_ (**a**), OleT_JH_ (**b**), OleT_SQ_ (**c**), and OleT_SA_ (**d**). The hydroxylation (HD) activity was calculated by subtracting the percentage of 1-alkene production from the total substrate conversion ratio. Results are shown as mean ± SD of two parallel experiments. In a standard assay, 1 μM of each OleT enzyme, 200 μM fatty acid substrate (C_8_–C_20_ FFAs), and 220 μM H_2_O_2_ were co-incubated in a 200-μL reaction system. The reactions were carried out at 30 °C for 2 h

Using purified OleT enzymes, we determined their activities towards a range of straight-chain saturated fatty acids (C_8_–C_20_) with H_2_O_2_ as cofactor. As results, all the four enzymes exhibited similar substrate preference profiles with decanoic acid (C_10_) or lauric acid (C_12_) as their optimal substrate (Fig. 3). The lauric acid conversion ratios were 93.8 ± 6.1%, 98.6 ± 0.6%, 99.1 ± 0.2%, and 86.2 ± 1.9% for OleT_JE_, OleT_JH_, OleT_SQ_, and OleT_SA_, respectively. With respect to the productivity of 1-alkenes, lauric acid was the best substrate unanimously. Of note, OleT_JE_, OleT_JH_, and OleT_SQ_ exhibited higher decarboxylation (DC) activities than hydroxylation (HD) activities with the DC/HD values up to 38.3 when C_10_–C_14_ FFAs were used as substrates. However, OleT_SA_ only showed moderately higher alkene production from its optimal substrates lauric acid (DC/HD = 2.6) and myristic acid (DC/HD = 4.0). By contrast, such chemoselectivity was entirely reversed when the long-chain FFA arachidic acid (C_20_) was the substrate, suggesting that substrates with different carbon-chain length could adopt distinct substrate-binding modes, thereby impacting the preference for decarboxylation or hydroxylation.

Subsequently, we elected to perform more detailed analyses of the myristic acid conversions catalyzed by all four OleT enzymes for two reasons: (1) myristic acid gave the highest DC/HD values among the tested FFAs, thus being the optimal decarboxylation substrate; (2) the commercial availability of α- and β-hydroxy myristic acid would enable quantification of the hydroxylation products. As expected, OleT_JE_, OleT_JH_, OleT_SQ_, and OleT_SA_ all produced 1-tridecene as the dominant product. As for hydroxylation products, β-OH-myristic acid was the major hydroxylation product (6.8–15.4% of the total products), α-OH-myristic acid only accounted for less than 1% of the total products, and no γ-, δ-, or ε-hydroxylated products were detected (Table 1). Taken together, OleT_JH_, OleT_SQ_, and OleT_SA_ are three new OleT_JE_-like FADCs, which can sometimes outperform the decarboxylation activity of OleT_JE_, depending on the FFA substrates to be decarboxylated.

**Table 1:** GC–MS analysis of substrate conversions and product distribution profiles of the four FADCs using myristic acid (C_14_) as substrate

Enzymes	Conversion (%)	Product distribution (%)		
		1-Tridecene	α–OH–C_14_	β–OH–C_14_
OleT_JE_	67.1 ± 2.5	91.7 ± 0.5	0.6 ± 0.1	7.8 ± 0.4
OleT_JH_	69.2 ± 4.6	92.9 ± 1.3	0.3 ± 0.1	6.8 ± 1.4
OleT_SQ_	62.6 ± 1.7	91.1 ± 0.5	0.2 ± 0.1	8.8 ± 0.5
OleT_SA_	60.7 ± 10.3	84.0 ± 6.5	0.6 ± 0.3	15.4 ± 6.2

To further quantitatively evaluate the catalytic efficiencies of OleT_JH_, OleT_SQ_, and OleT_SA_, we determined their steady-state kinetic parameters (Table 2) towards the optimal substrate lauric acid (C_12_) with OleT_JE_ as a control (Additional file 1: Figure S4). By measuring the initial substrate consumption rates with gas chromatography, we found that the kinetic parameters of OleT_JH_ were mostly similar to those of the control FADC OleT_JE_ with a slightly higher catalytic activity (*k*_cat_). In comparison, OleT_SQ_ and OleT_SA_ displayed relatively lower catalytic efficiency (*k*_cat_/*K*_*m*_) due to their attenuated *k*_cat_ values, albeit a slightly higher lauric acid substrate-binding affinity reflected by their lower *K*_*m*_. This may seem inconsistent with the previous results that OleT_SQ_ showed higher conversion rate (99.1 ± 0.2%) towards lauric acid than OleT_JE_ (93.8 ± 6.1%). We reason this contradiction might be that OleT_SQ_ could be more likely prone to H_2_O_2_ inactivation when used at low enzyme concentrations as in the kinetic studies.

**Table 2:** Steady-state kinetic parameters and reaction turnover numbers (TONs) of the four OleT enzymes

Enzymes	Kinetic parameters			TON^a^	TON^b^
	*k*_cat_ (min^−1^)	*K*_*m*_ (μM)	*k*_cat_*/K*_*m*_ (min^−1^ μM^−1^)		
OleT_JE_	860 ± 28	41 ± 4	21	95	185
OleT_JH_	910 ± 113	46 ± 15	20	100	180
OleT_SQ_	570 ± 55	35 ± 10	16	53	144
OleT_SA_	600 ± 13	37 ± 7	16	38	94

^a^H_2_O_2_-batch: 1 mM H_2_O_2_, 1 mM substrate (lauric acid), 1 μM purified OleT, 30 °C, 12 h

^b^H_2_O_2_ fed-batch: 5 times H_2_O_2_ addition (5 × 200 μM) every 2 h, 1 mM substrate (lauric acid), 1 μM purified OleT, 30 °C, 12 h; mean values and standard errors from two parallel experiments

Next, we evaluated the reaction turnover numbers (TONs) of the four FADCs at a higher substrate concentration (1 mM) for longer reaction time (12 h). A previous study showed that the fed-batch addition of H_2_O_2_ reduced enzyme inactivation and improved catalytic conversion [22]. We thus performed the assay with one-off and fed-batch supplementation of H_2_O_2_, respectively. As expected, the fed-batch addition of H_2_O_2_ resulted in higher TONs in general (Table 2). The maximum TON of 185 was obtained from OleT_JE_ while OleT_JH_ showed the best TON in 1 mM H_2_O_2_. OleT_JE_ and OleT_JH_ seemed to be relatively more resistant to H_2_O_2_ inactivation than OleT_SA_ and OleT_SQ_, as more than twofold (2.5 and 2.7) TON reductions were observed in H_2_O_2_ batch addition assays of the latter two enzymes compared with their fed-batch addition assays. Nevertheless, the three new FADCs exhibited strong decarboxylation potentials and similar kinetic parameters to OleT_JE_. Future reaction process optimization such as in situ generation of H_2_O_2_ [33, 34] or utilization of a redox cascade system [22, 26, 31] could be considered to attenuate the catalyst inactivation by high concentration H_2_O_2_.

### Effects of salt concentration on OleT enzymes

**Fig. 4:**
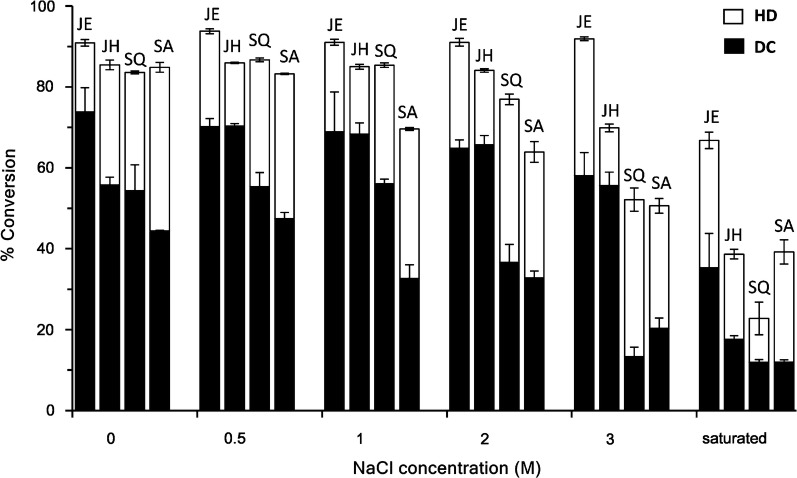
Effects of different NaCl concentrations on the activities of OleT_JE_ (JE), OleT_JH_ (JH), OleT_SQ_ (SQ) and OleT_SA_ (SA). The hydroxylation (HD) activity was calculated by subtracting the percentage of 1-alkene production from the total substrate conversion ratio. Results are shown as mean ± SD of two parallel experiments. In a standard assay, 1–3 μL of each OleT enzyme (stored in the storage buffer) was used in 200 μL reaction buffer containing different NaCl concentrations and 200 μM lauric acid (C_12_) as substrate

Considering the facts that precipitation of OleT_JE_ occurs in low salt buffer [28] and that the OleT enzyme host strains including *Jeotgalicoccus*, *Salinicoccus*, and *Staphylococcus* species are often associated with the property of halophilicity or salt tolerance [35–38], we investigated the effects of salt concentration on the activity and stability of the OleT enzymes. Experimentally, the activities of OleT_JE_, OleT_JH_, OleT_SQ_, and OleT_SA_ against lauric acid were evaluated under a range of NaCl concentrations. The overall and decarboxylation activities of all four enzymes remained almost unchanged (> 90%) within up to 2 M salt concentration (Fig. 4). However, when NaCl concentration was 3 M or higher, the activities of all enzymes decreased gradually to different extents, with the only exception of OleT_JE_ at 3 M NaCl. However, they all remained at least 22.7% activity in saturated salt concentration, even up to 66.8% for OleT_JE_.

Next, we examined the salt stability of these OleT enzymes. Upon a 12-h treatment with different salt solution buffers. It is evident that OleT_JE_ and OleT_JH_ showed much better stabilities than OleT_SQ_ and OleT_SA_ under all tested NaCl concentrations. Again, OleT_JE_ and OleT_JH_ were even able to retain a majority of overall and decarboxylation activities at the saturated concentration (Table 3). Overall, the majority of activity of all four enzymes was maintained in buffer with a NaCl concentration of 0.5–2 M. According to the most widely used standards for halophilicity by Kushner, i.e., extreme halophiles (best in 2.5–5.2 M salt), borderline extreme halophiles (best in 1.5–4.0 M salt), and moderate halophiles (best in 0.5–2.5 M salt) [39–42], which can also be applied to halophilic enzymes [43, 44], these P450 FADCs belong to moderate halophilic enzymes.

**Table 3:** Relative activities of the OleT enzymes after a 12-h treatment at 30 °C in storage buffer with different NaCl concentrations

Enzymes	Relative activities, %			
	0 M^c^	0.5 M	2 M	Saturated^d^
OleT_JE_^a^	84 ± 5	104 ± 2	97 ± 3	75 ± 2
OleT_JH_^a^	77 ± 3	102	102	79 ± 11
OleT_SQ_^a^	61 ± 3	93 ± 2	65 ± 6	30 ± 6
OleT_SA_^a^	57 ± 5	98 ± 4	85 ± 3	56 ± 4
OleT_JE_^b^	74 ± 2	95 ± 3	83 ± 2	67 ± 6
OleT_JH_^b^	51 ± 6	100 ± 6	90 ± 1	62 ± 8
OleT_SQ_^b^	56 ± 6	112 ± 3	68 ± 4	21 ± 1
OleT_SA_^b^	39 ± 10	91 ± 23	90 ± 3	66 ± 12

^a^Relative % conversion of C_12_ in comparison to the conversion ratio in the standard reaction buffer without pretreatment

^b^Relative % of C_11_ 1-alkene production in comparison to the conversion ratio in the standard reaction buffer without pretreatment

^c^“0” means that 1–3 μL OleT enzymes (in storage buffer) were used in a 200 μL NaCl-free reaction buffer

^d^“Saturated” means an at least 5 M NaCl concentration

### Site-directed mutagenesis of OleT_SA_ fatty acid decarboxylase

**Fig. 5:**
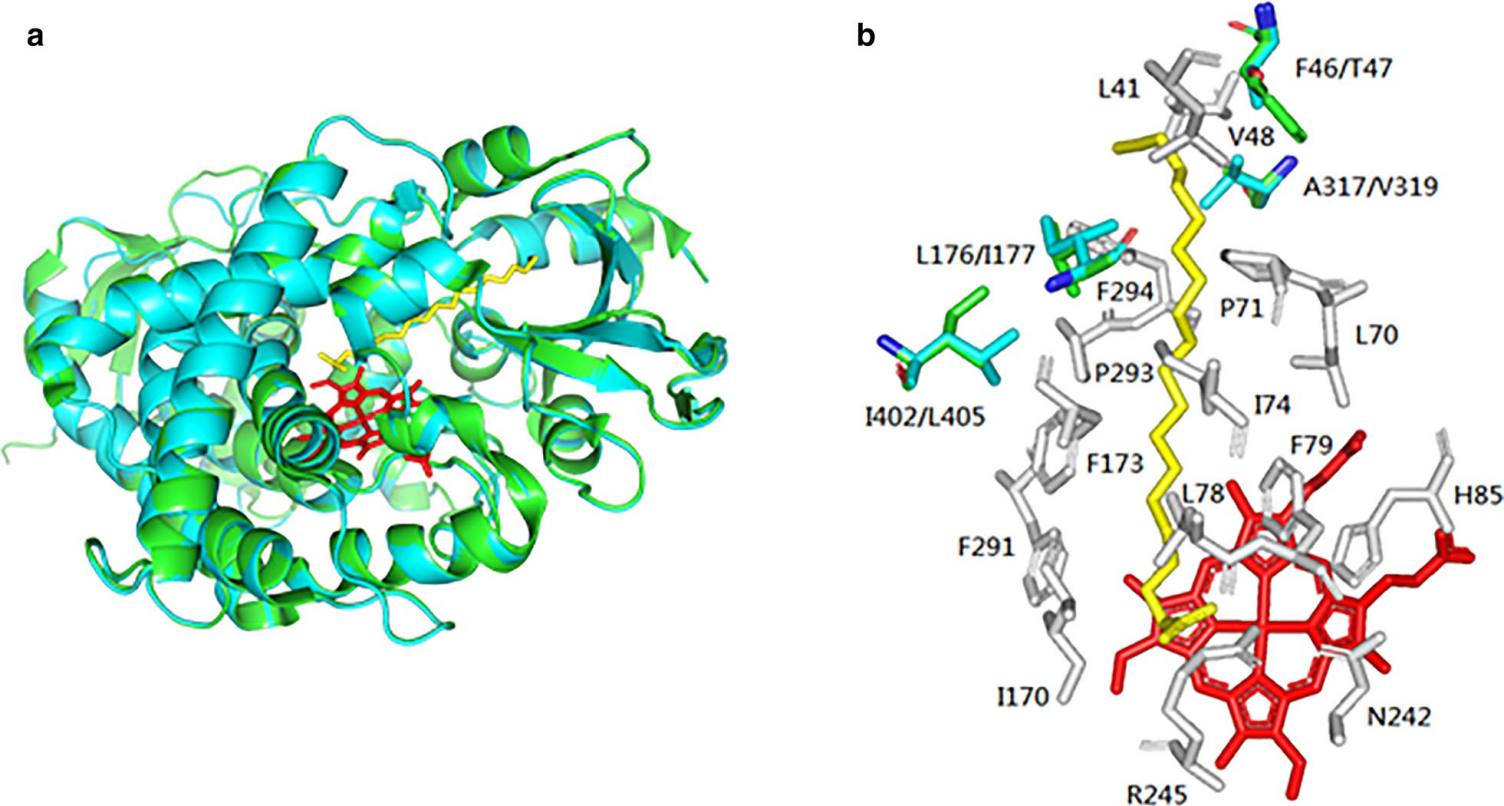
Comparison of overall structures and the substrate-binding pockets of OleT_SA_ and OleT_JE_. **a** The crystal structure of C20:0 fatty acid-bound OleT_JE_ (PDB: 4L40) is shown in green and the modeled structure of OleT_SA_ using Phyre^2^ software in cyan. **b** Comparison of substrate-binding pockets between OleT_JE_ (in green) and OleT_SA_ (in cyan). Red: heme; yellow: eicosanoic acid; gray: the same active site residues in OleT_JE_ and OleT_SA_

Despite significant advances in understanding of the OleT_JE_ decarboxylation mechanism [20, 23, 28, 29, 31, 45, 46], an effective strategy for engineering a better P450 FADC remains unclear. In this study, the four analogous OleT enzymes with characterized substrate specificity, product distribution and kinetic parameters provided an outstanding opportunity to further dissect the residues important for the decarboxylation activity. Specifically, we discovered, by comparing the crystal structure of OleT_JE_ (PDB ID #: 4L40) and the three modeled structures of the new FADCs, that OleT_JE_, OleT_JH_, OleT_SQ_, and OleT_SA_ have highly similar substrate-binding pockets with only five out of 19 residues that are different from one another. Comparatively, OleT_JE_ and OleT_SA_ have the most different substrate-binding pockets (Fig. 5 and Additional file 1: Table S2), which is consistent with their largest difference in catalytic activities among the four tested OleT enzymes towards mid-chain FFA substrates (C_8_–C_12_) (Fig. 3 and Table 2).

**Fig. 6:**
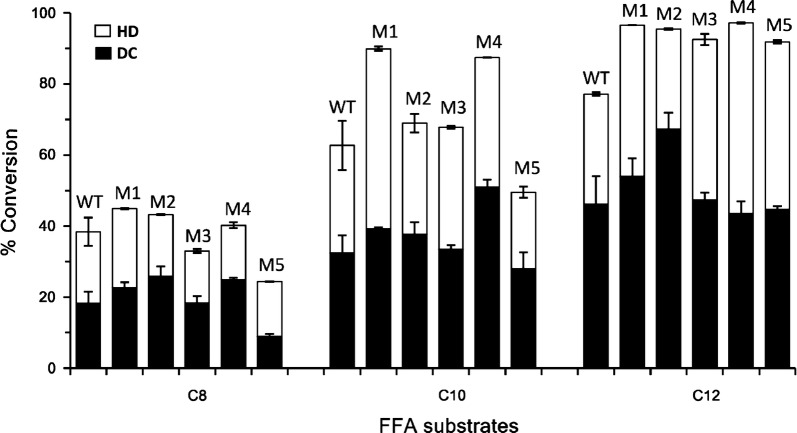
Comparison of the decarboxylation (DC) and hydroxylation (HD) activities between OleT_SA_ (WT) and its mutants. The C_8_–C_12_ FFAs were used as substrates for wild-type OleT_SA_ (WT) and the mutants including T47F (M1), I177L (M2), V319A (M3), L405I (M4) and T47F-I177L-V319A-L405I (M5). The hydroxylation (HD) activity was calculated by subtracting the percentage of 1-alkene production from the total substrate conversion ratio. Results are shown as mean ± SD of two parallel experiments

Following these analyses, we were drawn to mutate Thr47, Ile177, Val319, and Leu405 of OleT_SA_ into their counterparts in OleT_JE_ in consideration of the highest catalytic efficiency of OleT_JE_ and the most different catalytic activities and substrate-binding pockets between these two FADCs (Additional file 1: Table S2). Specifically, we prepared four single mutants including OleT_SA_–T47F (M1), OleT_SA_–I177L (M2), OleT_SA_–V319A (M3) and OleT_SA_–L405I (M4), and one quadruple mutant OleT_SA_–T47F-I177L–V319A–L405I (M5). Measurements of the in vitro activities of these mutants towards C_8_–C_12_ FFAs (Fig. 6 and Additional file 1: Figure S7) showed that (1) for caprylic acid (C_8_), M1, M2, and M4 displayed enhanced overall and decarboxylation activities, while M3 and M5 exhibited decreased overall and decarboxylation activities when compared to the wild-type OleT_SA_; (2) for decanoic acid (C_10_), all four single mutants showed improved overall and decarboxylation activities relative to their parental enzyme, but the activity of the quadruple mutant decreased; and (3) for lauric acid (C_12_), all mutants demonstrated > 20% improvement of substrate conversion ratios compared to the wild-type OleT_SA_. However, except for M2, other mutations did not lead to higher levels of 1-undecene (C_11_) production.

Collectively, all four tested positions could become the “hot spots” for the future iterative saturation mutagenesis (ISM) and combinatorial active-site saturation testing (CASTing) analyses [47, 48]. Here, the attenuated activity of OleT_SA_–T47F–I177L–V319A–L405I suggests there might not be an expected synergistic effect between these four residues.

## Discussion

In this study, we have expressed, purified and characterized three new P450 FADCs in vitro, namely, OleT_JH_, OleT_SQ_, and OleT_SA_ that can efficiently catalyze the conversions from FFAs to 1-alkenes. Among all substrates (C_8_–C_20_ FFAs), these three new enzymes exhibit higher decarboxylation activity towards lauric acid and myristic acid, with the lower decarboxylation activity for eicosanoic acid. The strict carbon chain length dependence for decarboxylative activity is consistent with the well-characterized FADCs OleT_JE_ [11] and CYP-Sm46∆29 [12], which further emphasize the importance of substrate identity in achieving the desirable reaction type. Our finding that β-hydroxyl product rather than α-hydroxyl product is the major by-product in all alkene-generating catalysis here supports the notion that C_β_–H abstraction may be one of the key factors that affect the production of 1-alkenes, which is well in line with the proposed mechanism by Makris et al. that Compound I (an iron(IV)-oxo cation radical) abstracts the C_β_–H atom to initiate the decarboxylation of fatty acids [20].

In general, salt solutions could impact protein folding mode, and hence the conformational stability of proteins [49–51]. We found that OleT_JH_, OleT_SQ_, OleT_SA_ and OleT_JE_ exhibit optimal activities as well as stabilities in salt solutions with the NaCl concentration ranging from 0.5 to 2 M (2.9–11.7%), thus classifying these FADCs as moderate halophilic enzymes. This seems to be consistent with the halophilic property of their native host microorganisms which have shown optimal growth at the NaCl strength of 2–3% (*Jeotgalicoccus halophilus*) [38], 3% (*Salinicoccus qingdaonensis*) [37] and 2.1–7% (*Staphylococcus aureus*) [52], respectively, thus being classified as moderate halophilic bacterial strains.

However, not all halophiles bear halophilic (P450) proteins. Two other P450 enzymes CYP102A26 [53] and CYP116B62 [54] from the halophilic bacteria *Pontibacillus halophilus* and *Halomonas* sp. NCIMB 172 exhibited decreased enzymatic activity in the presence of NaCl at 0.025 M and 0.05 M, respectively. This difference may be related to the different strategies how halophiles adapt to high salinity (“salt in” or “salt out”). Most halophilic bacteria use a “salt out” strategy to exclude salt to protect their non-halophilic enzymes [40]. While in a ‘salt in’ strategy that is used primarily by haloarchaea to accumulate high concentrations of salt, their enzymes have been examined to tolerate or even require 4–5 M salt [41].

Previous studies suggested that hydrogen bonds between negatively charged side chains and water molecules are critical for halophilic proteins to maintain a stable hydration shell under water-limited conditions [55, 56]. In a detailed study comparing 15 pairs of homologous non-halophilic and halophilic proteins (γ-glutamyltranspeptidases), it was shown that halophilic proteins tend to have increased number of acidic amino acids in their total surface residues, and within the whole protein sequences [57]. Thus, the ratio of acidic amino acids (Glu/Asp) in the total protein sequence of a halophilic protein has been considered as an important parameter to distinguish it from non-halophilic proteins from the same class of enzymes [39–41]. Notably, this is the first time that ‘halophilic P450 enzymes’ has been proposed for the need of salt to maintain the enzyme activity and stability. We subsequently calculated the ratios of acidic amino acids (Glu/Asp) in the total protein sequences for the four P450 halophilic FADCs. An average ratio of 14% (OleT_JE_: 14.5%, OleT_JH_: 14.2%, OleT_SQ_: 14.6%, OleT_SA_: 14.5%) (Figure S8) was obtained, which is comparable to a halophilic alkaline phosphatase (TAP: 14.0%) from *Halomonas* sp. 593 [58] and significantly higher than those non-halophilic P450 enzymes such as P450_BSβ_ (11.7%) and P450_SPα_ (11.8%) [14, 17]. However, detailed salt-tolerance mechanisms used by these P450s have yet to be elucidated. Furthermore, we suggest that a halophilic bacterial host for P450 FADCs could be engineered for efficient production of 1-alkenes based on the halophilic property of these CYP152 peroxygenases.

In our mutagenesis analyses, according to the catalytic activity profiles of the OleT_SA_ mutants, T47F mutation seemed to show more impact on the conversion of longer-chain FFAs (a 1.3-fold increase toward C_12_) than on that of the shorter chain C8 fatty acid (1.2-fold increase). Considering this residue is located at the top (the alkyl end) of the binding pocket, the bulky nature of the Phe side chain may help push an improved docking and binding of the longer chain fatty acid substrates. However, it is too far to the active site to make an impact on the chemo-selectivity of the enzymes (Fig. 5 and Additional file 1: Figure S7). Another mutation I177L is located in a structurally disordered region, the F–G loop. This distal loop area has been recently studied by Makris et al. suggesting its necessity for both substrate positioning and product release [45]. A single amino acid change at this site (L176G) switched OleT_JE_ from a decarboxylase to a hydroxylase [45]. Our results with the mutant OleT_SA_-I177L displayed improved catalytic conversion as well as the DC over HD selectivity towards all tested substrates, in particular the mid-chain fatty acid C_12_ (Additional file 1: Figure S7), demonstrating the crucial role of this leucine residue in OleT enzymes for the decarboxylation reaction. After all, the Ile to Leu replacement is only a minor structural change; the data suggest a delicate and accurate substrate positioning is required for regio- and chemo-selectivity of these P450 FADCs. As for the mutation L405I, since it is more into the middle and towards the bottom (the acyl end) of the binding pocket, the mutant OleT_SA_-L405I exhibited more prominent effect on the DC/HD selectivity towards the shorter-chain C_8_ fatty acid than by the other mutants (Additional file 1: Figure S7). However, despite the overall modulation of these mutants on the catalytic conversion and reaction selectivity of OleT_SA_, none of them are superior to OleT_JE_ in catalytic activity or decarboxylation selectivity. It was also difficult to see a synergistic effect from the quadruple mutant (Fig. 6).

These results together with previous studies [12, 24–26, 31], suggest that it remains challenging to rationally engineer a better substrate-binding pocket in OleT enzymes for better overall and decarboxylation activities. The residues that are distant from the substrate-binding site may be worth more attention in the future, which apparently requires a high-throughput screening (HTS) assay to enable the directed evolution [59–61] efforts. The development of such a HTS method is currently ongoing in our laboratory. Better unnatural FADCs would not only benefit the mechanistic understanding, but also increase the opportunity for industrialization of the intriguing α-olefin-producing enzymes.

## Conclusions

In this study, we biochemically characterized three new P450 FADCs that are able to efficiently decarboxylate a range of saturated fatty acids (C_8_–C_20_), with OleT_JH_ showing the decarboxylation activities, kinetic parameters, as well as salt tolerance and stability that are comparable to OleT_JE_. All four tested P450 FADCs exhibited moderate halophilicity unanimously. Further mutagenesis analysis based on protein sequence and activity comparisons of OleT_JE_ and OleT_SA_ provides more insights into the unique catalytic mechanism of P450 FADCs. The results lay out an advanced foundation for the future engineering efforts on this class of moderate halophilic P450 enzymes in order to produce desirable carbon chain length 1-alkenes efficiently.

## Methods

### Materials

The strains of *E. coli* DH5α and BL21(DE3) were preserved by our laboratory. All chemicals and antibiotics were obtained from TCI (Shanghai, China), Solarbio (Beijing, China), Sigma Aldrich (St. Louis, MO, USA) or Thermo Scientific (Shanghai, China). I-5™ 2× High-Fidelity Master Mix and Trelief SoSoo Cloning Kit were obtained from TsingKe (Beijing, China). Plasmid Miniprep Kit from TsingKe was used to prepare plasmid DNA from *E. coli* DH5α. ClonExpress II One-Step Cloning Kit was purchased from Vazyme (Nanjing, China). The 10× QuickRun™ Fast Running Buffer and FlexiRun™ premixed gel solution for SDS-PAGE were obtained from MDBio (Xinbei, China). Ni–NTA resin used for protein purification was supplied by Sangon Biotech (Shanghai, China). PD-10 desalting columns were purchased from GE Healthcare (Piscataway, NJ, USA). Millipore Amicon Ultra centrifugal filters were obtained from Millipore (Billerica, MA, USA).

### Bioinformatics analysis

The CYP152 family protein sequences were BLAST searched against the protein database on National Center for Biotechnology Information (NCBI) based on the query sequences of OleT_JE_ (CYP152L1) and CYP-Sm46Δ29 (CYP152L2). Proteins with a sequence identity greater than 60% were selected and the sequence alignments were performed using ClustalW (https://www.genome.jp/tools-bin/clustalw). The phylogenetic tree was built using the neighbor-joining (NJ) method in MEGA 7.0 package. Bootstrap values shown next to the branches were computed from 1000 bootstrap tests.

### Molecular cloning and protein purification

The gene sequences encoding OleT_JH_ from *Jeotgalicoccus halophilus* (CYP152L1_ortholog, GenBank accession number: WP_092595307), OleT_SQ_ from *Salinicoccus qingdaonensis* (CYP152L8, GenBank accession number: WP_092983663), and OleT_SA_ from *Staphylococcus aureus* (CYP152L7, GenBank accession number: WP_049319149) were codon-optimized and synthesized by Qinglan (Yixing, China), and then cloned into the vector pET28b via the *Nde*I/*Xho*I restriction sites for expression of the N-terminal His_6_-tagged proteins. The generation of single mutation gene constructs of OleT_SA_–T47F, OleT_SA_–I177L, OleT_SA_–V319A, OleT_SA_–L405I was achieved by site-directed mutagenesis via overlap extension PCR [62]. The sequences of primers used in this study are listed in Additional file 1: Table S1). All cloned sequences were confirmed by DNA sequencing at Sangon Biotech (Shanghai, China), and then used to transform *E. coli* BL21 (DE3) for protein expression and purification.

The *E. coli* BL21 (DE3) cells carrying the recombinant expression vector were grown 12 h at 37 °C with shaking at 220 rpm and then used as seed cultures to inoculate (1:100 ratio) a modified Terrific Broth medium containing 4% glycerol, a rare salt solution [26] and 1 mM thiamine. Cells were grown at 37 °C for 3–4 h until the optical density at 600 nm (OD_600_) reached 0.8–1.0, to which δ-aminolevulinic acid (5-ALA, 0.5 mM) and isopropyl β-d-1-thiogalactopyranoside (IPTG, 0.2 mM) were added, and followed by 24 h of cultivation at 18 °C. The cells were harvested (6000 ×*g*, 4 °C, 10 min) and then stored at − 80 °C.

Purification of His-tagged OleT enzymes was carried out as described by Liu et al. [26] with slight modifications. The cell pellets stored at − 80 °C were taken out to melt at room temperature, then all the following steps were performed at 4 °C. Briefly, the cell pellets were re-suspended in 50 mL lysis buffer (pH 8.0, 50 mM NaH_2_PO_4_, 300 mM NaCl, 10% glycerol, 10 mM imidazole) through vortexing and were then disrupted by ultrasonication. Cell-free lysate was obtained by centrifuging at 10,000 ×*g* for 1 h at 4 °C, to which 1–2 mL Ni–NTA resin slurry was added and mixed gently for 2 h. The mixture was loaded onto an empty column and washed with approximately 200 mL wash buffer (pH 8.0, 50 mM NaH_2_PO_4_, 300 mM NaCl, 10% glycerol, 20 mM imidazole) until no protein was eluted in flow-through. His-tagged proteins bound to Ni–NTA resin were eluted with 10 mL elution buffer (pH 8.0, 50 mM NaH_2_PO_4_, 500 mM NaCl, 10% glycerol, 250 mM imidazole). The eluents were concentrated with an Amicon Ultra centrifugal filter (30 kDa cutoff) and were buffer-exchanged into storage buffer (pH 7.4, 50 mM NaH_2_PO_4_, 500 mM NaCl, 10% glycerol). The final purified proteins were flash frozen by liquid nitrogen and stored at − 80 °C for later use.

### UV–visible spectroscopic characterization and determination of enzyme concentration

The analysis of the UV–visible spectroscopic properties was carried out as described by Xu et al. with minor modifications [12]. In general, the UV–visible spectroscopic properties of the His-tagged proteins were performed on a Cary 60 UV–visible spectrophotometer (Varian, UK). For preparation of the dithionite-reduced ferrous-CO complex of each enzyme, the purified ferric enzymes were diluted in Storage buffer (pH 7.4, 50 mM NaH_2_PO_4_, 500 mM NaCl, 10% glycerol) and subjected to slow CO bubbling for 40–50 s for initial scan at 350–600 nm to record the spectrum of the resting-state CO-bound proteins, and then followed by sufficient reduction of the protein by sodium dithionate to obtain the CO-bound reduced difference spectrum. The protein concentration was calculated by the reduced differential extinction coefficient ε_450–490_ nm of 91,000 M^−1^ cm^−1^ for the functional P450 concentration [63].

### In vitro enzymatic assay

Typical assays containing 1 μM of each enzyme (OleT_JE_, OleT_JH_, OleT_SQ_, OleT_SA_ or its individual mutant), 200 μM fatty acid substrate (C_8_–C_20_ FFAs prepared from stock solution in DMSO (20 mM)), and 220 μM H_2_O_2_ in 200 μL of buffer (pH 7.4, 50 mM NaH_2_PO_4_, 500 mM NaCl, 10% glycerol) were carried out at 30 °C for 2 h. This amount of hydrogen peroxide was proven to be sufficient (Additional file 1: Figure S9). Reactions were quenched by adding 20 μL 10 M HCl, then heptadecanoic acid (C_17_) was added as internal standard and the mixture was extracted by 150 μL ethyl acetate. The organic phase was analyzed by gas chromatography (GC) as described below.

For detection of 1-heptene (C_7_) product generated from caprylic acid (C_8_) fatty acid decarboxylation, 1.5 mL polytetrafluorethylene (PTFE) septum-sealed glass bottles were used for 500 μL reaction systems containing 1 μM enzyme, 200 μM C_8_ fatty acid substrate, 220 μM H_2_O_2_, and 200 μM 1-nonene (C_9_) as the internal standard. The reactions were incubated at 30 °C for 2 h with shaking at 100 rpm. Then the reactions were placed at 4 °C for 12 h to stop reactions prior to heating at 40 °C for 20 min for headspace sampling using a gas-tight Hamilton syringe for GC–MS analysis. Different concentrations of the authentic 1-heptene standard incubated under the same conditions as reactions were analyzed using the same GC–MS method to obtain the standard curve. After headspace sampling, the remaining C_8_ substrate in reactions was extracted as above described for further GC measurement and analysis.

### Effects of salt concentration

Effect of salt (NaCl) was tested by measuring the decarboxylation activity in the reaction buffer (pH 7.4, 50 mM NaH_2_PO_4_, 10% glycerol) containing different concentrations of NaCl ranging from 0 M, 0.5 M, 1 M, 2 M, 3 M to saturated salinity (> 5 M) at 30 °C for 2 h. To determine their halostability, these enzymes were pre-incubated in buffer (pH 7.4, 50 mM NaH_2_PO_4_, 10% glycerol) containing different concentrations of NaCl (0 M, 0.5 M, 2 M, and saturated salt solution) at 30 °C for 12 h with non-treated enzyme as the control. The residual activities were then measured as in typical assays. For all reactions in salt effect experiments, 1–3 μL of enzymes stored at − 80 °C in storage buffer (pH 7.4, 50 mM NaH_2_PO_4_, 500 mM NaCl, 10% glycerol) were used in 200 μL reaction systems containing different concentrations of salt.

### Steady-state kinetic analysis

Determination of the steady-stated kinetic parameters was carried out as described by Xu et al. with minor modifications [12]. Briefly, 15–40 nM enzyme was used with a range of different substrate (C_12_) concentrations in a 1 mL reaction system (pH 7.4, 50 mM NaH_2_PO_4_, 500 mM NaCl, 10% glycerol). The reaction was initiated by adding an excess amount of H_2_O_2_ (330 μM) at 30 °C. Aliquots (200 μL) of reactions were removed and quenched at fixed time points (0, 0.5, 1, 2 min) by adding 20 μL 10 M HCl for reaction termination. Sample extraction and analysis were performed the same as above for GC analysis. Initial rates were calculated in terms of the substrate consumption by each enzyme. Kinetic analyses were performed using OriginPro 8.5 program.

### Homology modeling

The 3D structures of OleT_JH_, OleT_SQ_ and OleT_SA_ were generated by using the homology modeling function of Phyre^2^ (Protein Homology/analogy Recognition Engine V2.0) program. The structure of OleT_JE_ (PDB ID #: 4L40) and the modeled structures were analyzed with PyMOL (V2.2.0).

### Analytical methods

The hydrocarbon and fatty acid samples were analyzed by the methods from Guan et al. [64]. The Agilent 7890B gas chromatograph equipped with a capillary column HP-5 (Agilent Technologies, Santa Clara, CA, USA; cross-linked polyethylene glycerol, i.d. 0.25 μm film thickness, 30 m by 0.32 mm) was used for analyses. The flow rate of helium was set to 1 mL per min. The oven program was set initially at 40 °C for 4 min, then increased to 280 °C by the rate of 10 °C per min and held for 5 min. The injecting temperature was set to 280 °C under splitless injection conditions with 1 μL injection volume. Under these conditions, the retention times and signal intensity of fatty acids and terminal alkenes products were determined and compared with corresponding authentic standards (FFAs: C_8_–C_20_, 1-alkenes: C_7_–C_19_) and the internal standard (1-heptadecanoic acid (C_17_)). For analyses of GC–MS, the gas chromatography was equipped with an Agilent 5975C MSD single quadrupole mass spectrometer operated under electron ionization mode at 70 eV in the scan range of 50–500 *m/z*. For detection of α- and β-hydroxyl products of myristic acid, the samples extracted from the myristic acid (C_14_) reactions were derivatized with an equal volume of *N,O*-bis (trimethylsilyl) trifluoroacetamide (BSTFA) with 1% trimethylchlorosilane at 72 °C for 15 min before GC–MS analysis. The GC–MS analysis used the previous protocol adapted from Rude et al. with the Agilent J&W DB-5MS column (i.d. 0.25 μm film thickness, 50 m by 0.25 mm). Peak identity was determined by comparisons of the retention time and fragmentation pattern with the authentic standard compounds where available and to National Institute of Standards and Technology, USA mass spectral database. We found that the sum of all products (α-, β-hydroxy myristic acids and 1-tridecene) almost accounted for 99% of the substrate consumption, so we subtracted the 1-alkene production from the total substrate consumption to quantify the percentage of hydroxylated products for all substrates unless otherwise stated. For detection of 1-heptene (C_7_) product produced by caprylic acid (C_8_) decarboxylation, 500 μL of the reaction headspace sample was injected into GC–MS system with a Hamilton needle syringe and analyzed by authentic standard curves as well as reaction controls. The oven temperature procedure was set at follows: 40 °C for 2 min, then increased to 100 °C by 5 °C min^−1^ and held for 2 min.

## Additional files

**Additional file 1: Figure S1.** Protein sequence alignment of the P450 fatty acid decarboxylases OleT_JE_ from *Jeotgalicoccus* sp. ATCC 8456 (GenBank accession number: ADW41779) and CYP-Sm46Δ29 (with the N-terminal redundant 29 amino acids deleted) from *Staphylococcus massiliensis* S46 (GenBank accession number: WP_039990689), with the newly identified FADCs of OleT_JH_ from *Jeotgalicoccus halophilus* (GenBank accession number: WP_092595307), OleT_SQ_ from *Salinicoccus qingdaonensis* (GenBank accession number: WP_092983663), and OleT_SA_ from *Staphylococcus aureus* (GenBank accession number: WP_049319149). The orange stars indicate the residues (79F, 85H, 170I, 245R) that have been reported to be important for decarboxylation by P450 OleT_JE_. The blue stars indicate the key different amino acid residues. **Figure S2.** SDS-PAGE analysis of the purified His_6_-tagged OleT_JH_ (lane A), OleT_SQ_ (lane B), OleT_SA_ (lane C), and protein marker (M). **Figure S3.** UV-visible spectra of OleT_JE_ (A), OleT_JH_ (B), OleT_SQ_ (C) and OleT_SA_ (D). The purified enzymes were diluted in buffer (pH 7.4) containing 50 mM NaH_2_PO4, 500 mM NaCl and 10% glycerol. (Black lines show the spectra for the oxidized ferric form of CYPs and red lines show the spectra for the Na_2_S_2_O_4_-reduced ferrous-CO complex of CYPs; Insets exhibit the reduced CO-bound difference spectra of P450 enzyme). **Figure S4.** Kinetic curves of OleT_JE_, OleT_JH_, OleT_SQ_ and OleT_SA_ against their optimal substrate (lauric acid) were fitted to Michaelis-Menten equation respectively. (A) Lauric acid (C_12_) substrate consumption rates by OleT_JE_; (B) Lauric acid (C_12_) substrate consumption rates by OleT_JH_; (C) Lauric acid (C_12_) substrate consumption rates by OleT_SQ_; (D) Lauric acid (C_12_) substrate consumption rates by OleT_SA_. The steady state kinetic parameters were calculated using OriginPro 8.5 and are summarized in Table 2. **Figure S5.** UV-visible spectra of the OleT_SA_ mutants: T47F (A), I177L (B), V319A (C), L405I (D) and T47F/I177L/V319A/L405I (E). The purified enzymes were diluted in buffer (pH 7.4) containing 50 mM NaH_2_PO_4_, 500 mM NaCl and 10% glycerol, respectively. (Black lines show the spectra for the oxidized ferric form of CYPs and red lines show the spectra for the Na_2_S_2_O_4_-reduced ferrous-CO complex of CYPs; Insets exhibit the reduced CO-bound difference spectra of P450 enzyme). **Figure S6.** SDS-PAGE analysis of the purified His_6_-tagged mutants, including T47F (lane A), I177L (lane B), V319A (lane C), L405I (lane D), T47F/I177L/V319A/L405I (lane E), and protein marker (M). **Figure S7.** Decarboxylation (DC) versus hydroxylation (HD) activities (ratios) of OleT_SA_ (WT) and its mutants including T47F (M1), I177L (M2), V319A (M3), L405I (M4), and T47F-I177L-V319A-L405I (M5) towards mid-chain fatty acids (C_8_–C_12_). **Figure S8.** Analysis of acidic amino acids (aspartic acid in red and glutamic acid in cyan) in the protein structure of OleT_JE_ (A: PDB ID #: 4L40) and the modeled protein structures by Phyre^2^ (B: OleT_JH_; C: OleT_SQ_; D: OleT_SA_). **Figure S9.** The effect of H_2_O_2_ concentration on substrate conversion ratios for four FADCs (A: OleT_JE_; B: OleT_JH_; C: OleT_SQ_; D: OleT_SA_) in our standard reaction system (200 μM lauric acid substrate, 1 μM purified OleT enzyme, 30 °C for 2 h). **Table S1.** Primers used for cloning and site-directed mutagenesis in this study. **Table S2.** Major differences in substrate-binding-site residues composition among OleT_JE_ and the three newly identified P450 FADCs.

**Additional file 2.** The codon-optimized gene sequences of OleT_JH_, OleT_SQ_ and OleT_SA_ and their corresponding amino acid sequences.

## Abbreviations

CYP or P450: cytochrome P450 enzyme
FFA: free fatty acid
FADCs: fatty acid decarboxylases
DC: decarboxylation
HD: hydroxylation
TON: turnover number
PCR: polymerase chain reaction
GC–MS: gas chromatography–mass spectrometry
