# Supplementary material for: Biochemical characterization of three new α-olefin-producing P450 fatty acid decarboxylases with a halophilic property
Source: Biotechnol Biofuels. 2019 Apr 8;12:79. doi: 10.1186/s13068-019-1419-6 (PMC6452516; doi:10.1186/s13068-019-1419-6)
Supplement: Supplementary file 1 — Additional file 1: Figure S1. Protein sequence alignment of the P450 fatty acid decarboxylases OleTJE from Jeotgalicoccus sp. ATCC 8456 (GenBank accession number: ADW41779) and CYP-Sm46Δ29 (with the N-terminal redundant 29 amino acids deleted) from Staphylococcus massiliensis S46 (GenBank accession number: WP_039990689), with the newly identified FADCs of OleTJH from Jeotgalicoccus halophilus (GenBank accession number: WP_092595307), OleTSQ from Salinicoccus qingdaonensis (GenBank accession number: WP_092983663), and OleTSA from Staphylococcus aureus (GenBank accession number: WP_049319149). The orange stars indicate the residues (79F, 85H, 170I, 245R) that have been reported to be important for decarboxylation by P450 OleTJE. The blue stars indicate the key different amino acid residues. Figure S2. SDS-PAGE analysis of the purified His6-tagged OleTJH (lane A), OleTSQ (lane B), OleTSA (lane C), and protein marker (M). Figure S3. UV-visible spectra of OleTJE (A), OleTJH (B), OleTSQ (C) and OleTSA (D). The purified enzymes were diluted in buffer (pH 7.4) containing 50 mM NaH2PO4, 500 mM NaCl and 10% glycerol. (Black lines show the spectra for the oxidized ferric form of CYPs and red lines show the spectra for the Na2S2O4-reduced ferrous-CO complex of CYPs; Insets exhibit the reduced CO-bound difference spectra of P450 enzyme). Figure S4. Kinetic curves of OleTJE, OleTJH, OleTSQ and OleTSA against their optimal substrate (lauric acid) were fitted to Michaelis-Menten equation respectively. (A) Lauric acid (C12) substrate consumption rates by OleTJE; (B) Lauric acid (C12) substrate consumption rates by OleTJH; (C) Lauric acid (C12) substrate consumption rates by OleTSQ; (D) Lauric acid (C12) substrate consumption rates by OleTSA. The steady state kinetic parameters were calculated using OriginPro 8.5 and are summarized in Table 2. Figure S5. UV-visible spectra of the OleTSA mutants: T47F (A), I177L (B), V319A (C), L405I (D) and T47F/I177L/V319A/L405I (E). The purified en [file 13068_2019_1419_MOESM1_ESM.pdf]

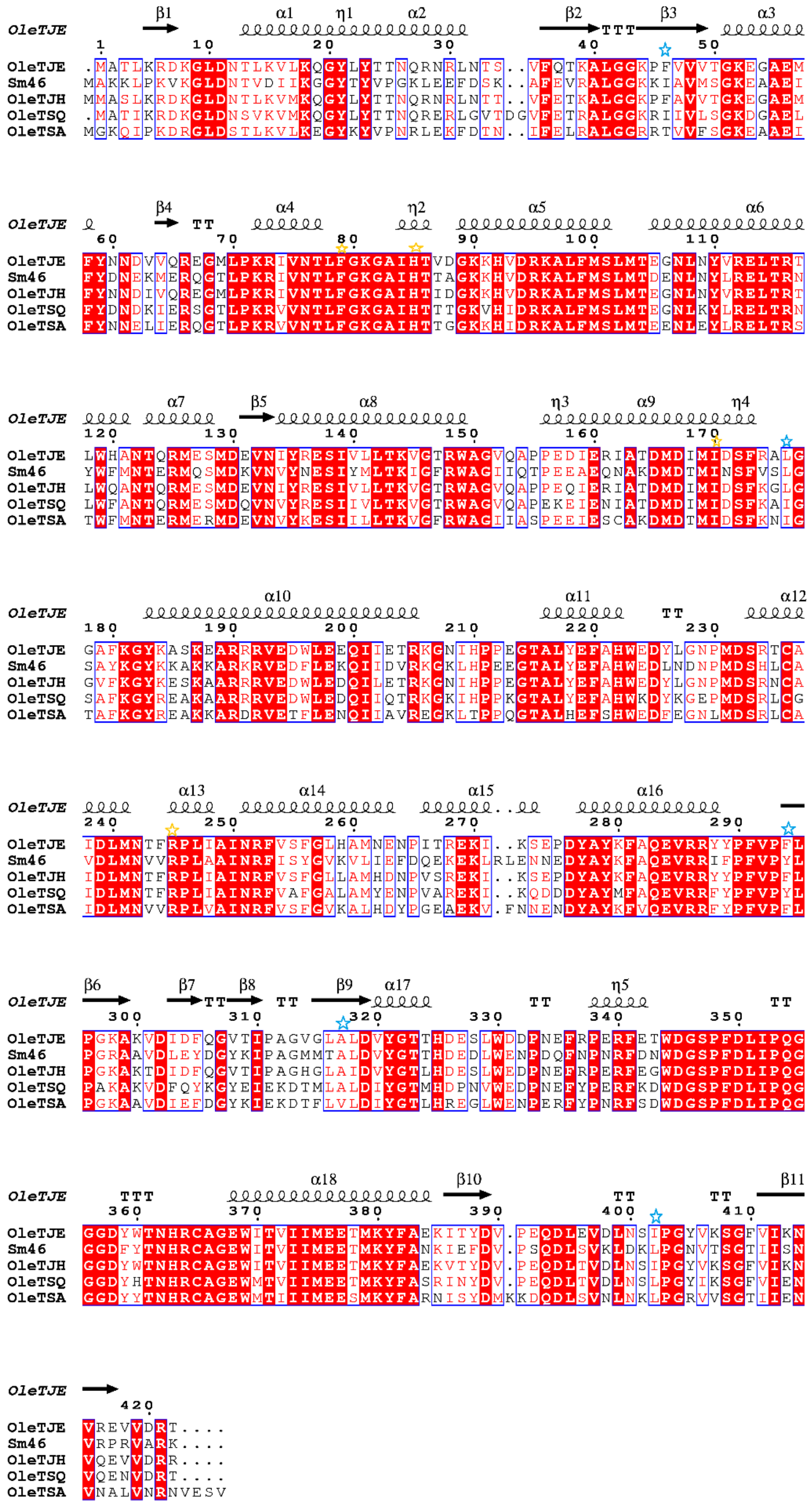

Figure S1. Protein sequence alignment of the P450 fatty acid decarboxylases OleT$_{JE}$ from *Jeotgalicoccus sp.* ATCC 8456 (GenBank accession number: ADW41779) and

CYP-Sm46Δ29 (with the N-terminal redundant 29 amino acids deleted) from *Staphylococcus massiliensis* S46 (GenBank accession number: WP_039990689), with the newly identified FADCs of $OleT_{JH}$ from *Jeotgalicoccus halophilus* (GenBank accession number: WP_092595307), $OleT_{SQ}$ from *Salinicoccus qingdaonensis* (GenBank accession number: WP_092983663), and $OleT_{SA}$ from *Staphylococcus aureus* (GenBank accession number: WP_049319149). The orange stars indicate the residues (79F, 85H, 170I, 245R) that have been reported to be important for decarboxylation by P450 $OleT_{JE}$. The blue stars indicate the key different amino acid residues.

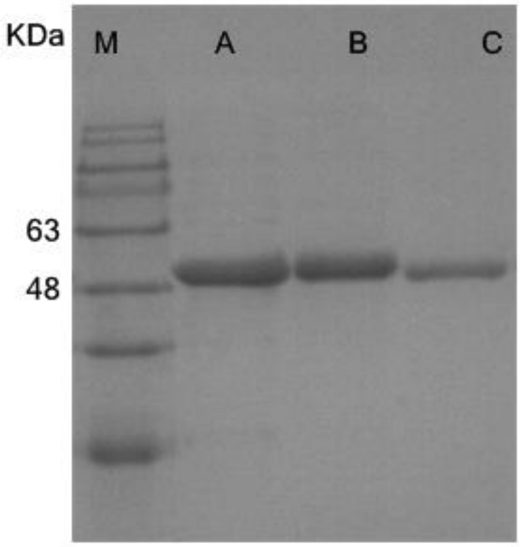

Figure S2. SDS-PAGE analysis of the purified His_6-tagged $OleT_{JH}$ (lane A), $OleT_{SQ}$ (lane B), $OleT_{SA}$ (lane C), and protein marker (M).

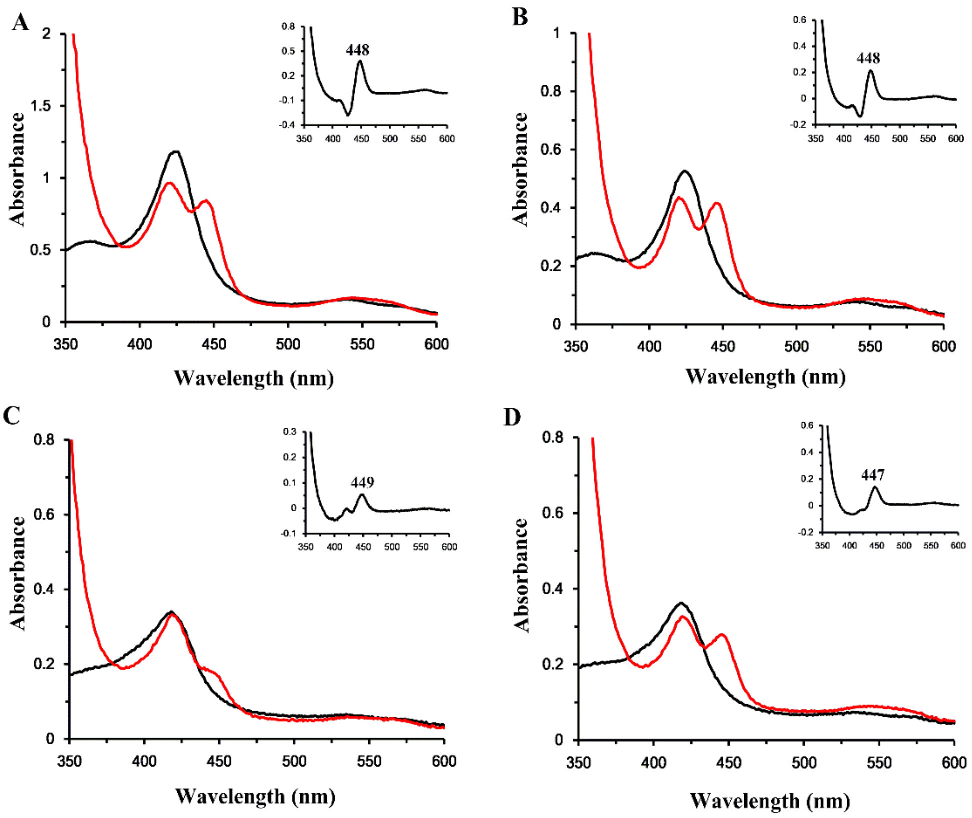

Figure S3. UV-visible spectra of OleT$_{JE}$ (A), OleT$_{JH}$ (B), OleT$_{SQ}$ (C) and OleT$_{SA}$ (D). The purified enzymes were diluted in buffer (pH 7.4) containing 50 mM NaH_2PO4, 500 mM NaCl and 10% glycerol. (Black lines show the spectra for the oxidized ferric form of CYPs and red lines show the spectra for the $Na_2S_2O_4$-reduced ferrous-CO complex of CYPs; Insets exhibit the reduced CO-bound difference spectra of P450 enzyme).

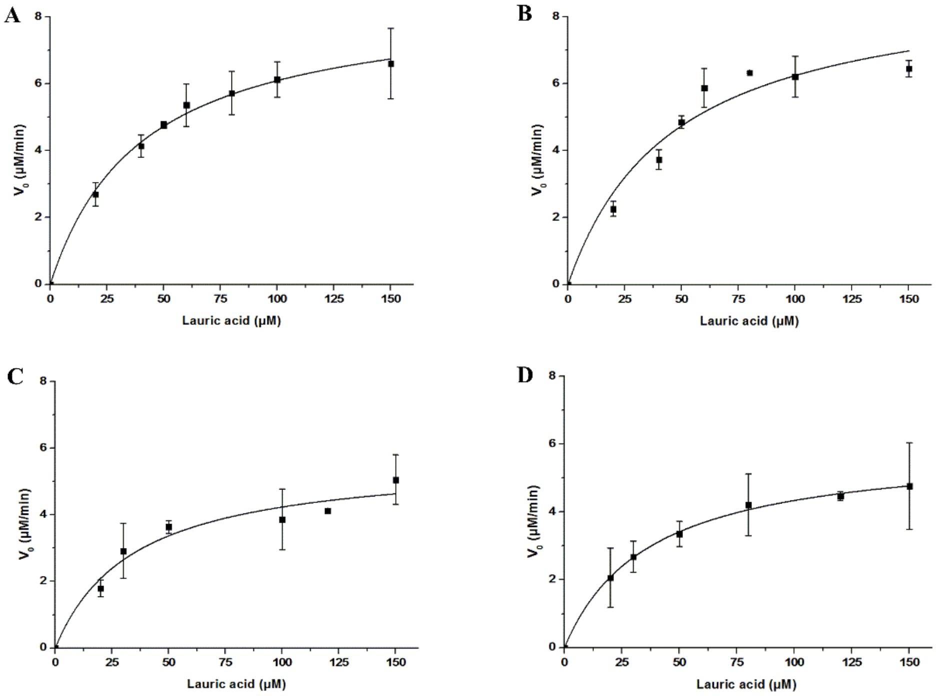

Figure S4. Kinetic curves of $OleT_{JE}$, $OleT_{JH}$, $OleT_{SQ}$ and $OleT_{SA}$ against their optimal substrate (lauric acid) were fitted to Michaelis-Menten equation respectively. (A) Lauric acid (C_{12}) substrate consumption rates by $OleT_{JE}$; (B) Lauric acid (C_{12}) substrate consumption rates by $OleT_{JH}$; (C) Lauric acid (C_{12}) substrate consumption rates by $OleT_{SQ}$; (D) Lauric acid (C_{12}) substrate consumption rates by $OleT_{SA}$. The steady state kinetic parameters were calculated using OriginPro 8.5 and are summarized in Table 2.

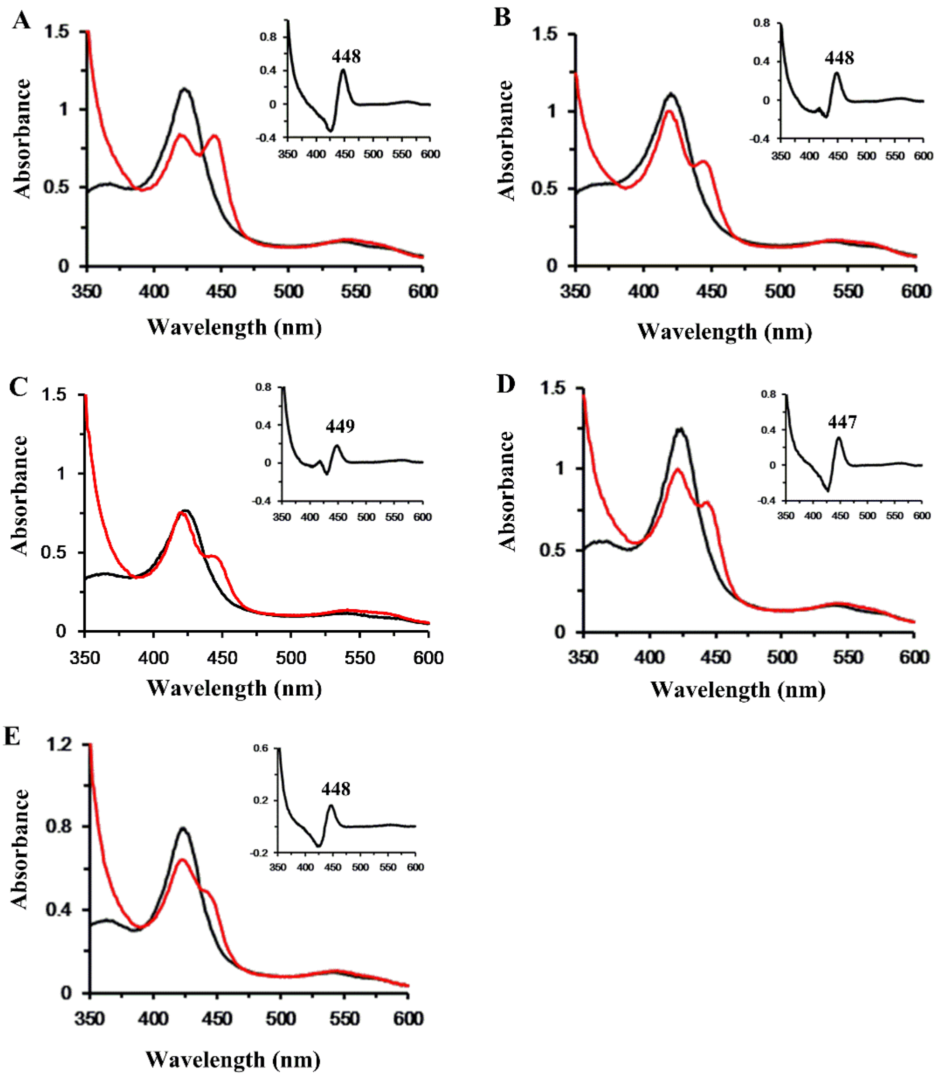

Figure S5. UV-visible spectra of the $OleT_{SA}$ mutants: T47F (A), I177L (B), V319A (C), L405I (D) and T47F/I177L/V319A/L405I (E). The purified enzymes were diluted in buffer (pH 7.4) containing 50 mM NaH_2PO_4, 500 mM NaCl and 10% glycerol, respectively. (Black lines show the spectra for the oxidized ferric form of CYPs and red lines show the spectra for the $Na_2S_2O_4$-reduced ferrous-CO complex of CYPs; Insets exhibit the reduced CO-bound difference spectra of P450 enzyme).

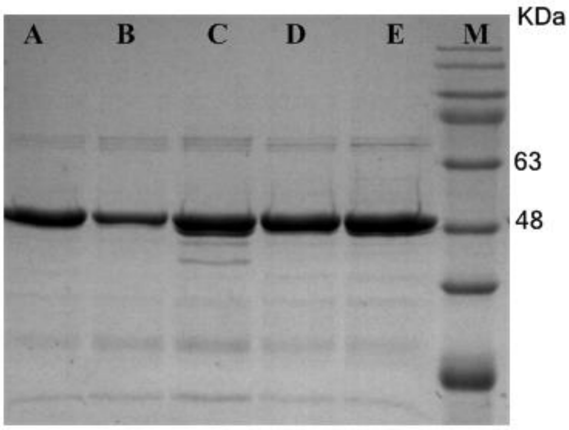

Figure S6. SDS-PAGE analysis of the purified His_6-tagged mutants, including T47F (lane A), I177L (lane B), V319A (lane C), L405I (lane D), T47F/I177L/V319A/L405I (lane E), and protein marker (M).

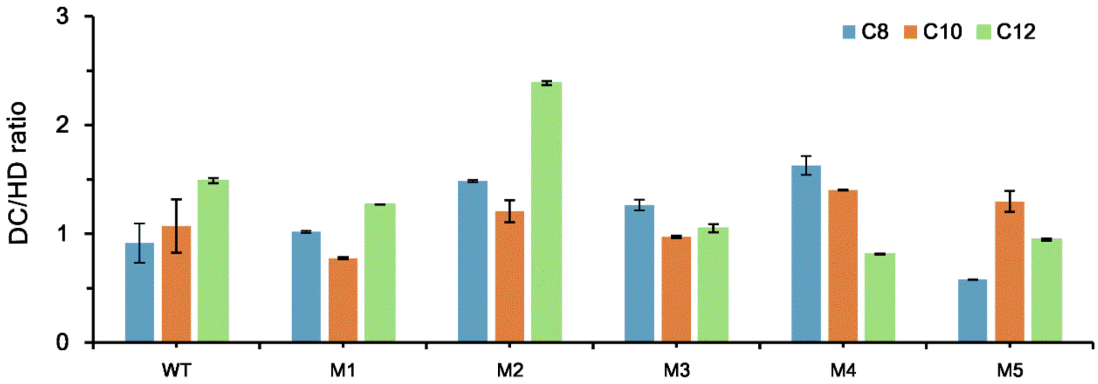

Figure S7. Decarboxylation (DC) *versus* hydroxylation (HD) activities (ratios) of $OleT_{SA}$ (WT) and its mutants including T47F (M1), I177L (M2), V319A (M3), L405I (M4), and T47F-I177L-V319A-L405I (M5) towards mid-chain fatty acids (C_8-C_{12}).

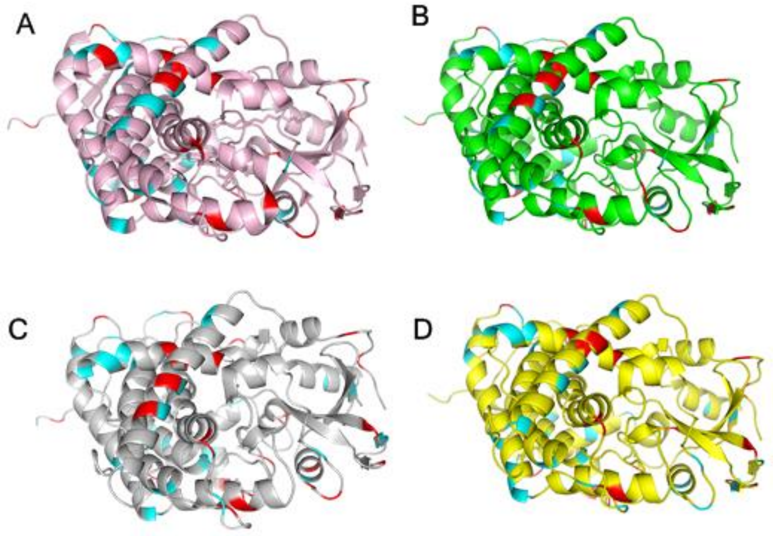

Figure S8. Analysis of acidic amino acids (aspartic acid in red and glutamic acid in cyan) in the protein structure of $OleT_{JE}$ (A: PDB ID #: 4L40) and the modeled protein structures by Phyre[2] (B: $OleT_{JH}$; C: $OleT_{SQ}$; D: $OleT_{SA}$).

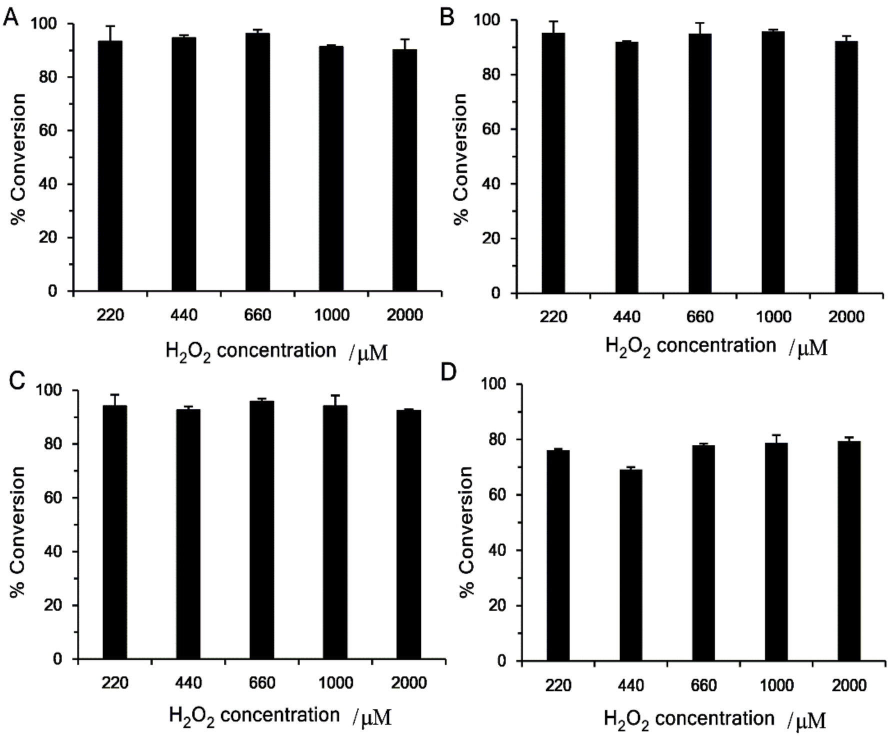

Figure S9. The effect of H_2O_2 concentration on substrate conversion ratios for four FADCs (A: $OleT_{JE}$; B: $OleT_{JH}$; C: $OleT_{SQ}$; D: $OleT_{SA}$) in our standard reaction system (200 μM lauric acid substrate, 1 μM purified OleT enzyme, 30 °C for 2 h).

Table S1. Primers used for cloning and site-directed mutagenesis in this study.

Primer	Sequence (5'→3')
$OleT_{JH}$-F	GGAATTC**CATATG**ATGGCGAGCCTGAAACGC
$OleT_{JH}$-R	CCG**CTCGAG**TTAGCGGCGATCCACCACTTCC
$OleT_{SQ}$-F	GGAATTC**CATATG**GCGACCATTAAACGCGAT
$OleT_{SQ}$-R	CCG**CTCGAG**TTAGGTGCGATCCACGTTTTCC
$OleT_{SA}$-F	GGAATTC**CATATG**GGCAAACAGATTCCGAAA
$OleT_{SA}$-R	CCG**CTCGAG**TTACACGCTTTCCACATTGCGG
$OleT_{SA}$-47-F	CGTCGTTTCGTTGTGTTTAGCGGCAAA
$OleT_{SA}$-47-R	CACAACGAAACGACGACCACCTAACGC
$OleT_{SA}$-177-F	AAAAACCTTGGCACCGCGTTTAAAGGC
$OleT_{SA}$-177-R	GGTGCCAAGGTTTTTGAAGCTGTCGAT
$OleT_{SA}$-319-F	TTCCTGGCACTGGATATTTATGGCACC
$OleT_{SA}$-319-R	ATCCAGTGCCAGGAAGGTATCCTTTTC
$OleT_{SA}$-405-F	AACAAAATCCCGGGCCGTGTTGTTAGC
$OleT_{SA}$-405-R	GCCCGGGATTTTGTTCAGGTTCACGCT
$OleT_{SA}$-47-177-319-405-F	GGAATTC**CATATG**GGCAAACAGATTCCGAAA
$OleT_{SA}$-47-177-319-405-R	CCG**CTCGAG**CACGCTTTCCACATTGCGGTTC

The bold nucleotides denote the restriction site of *Nde*I and *Xho*I. Protective bases are in blue. The red nucleotides denote the mutated codons and the sequences of base complementary are underlined.

Table S2. Major differences in substrate-binding-site residue composition among $OleT_{JE}$ and the three newly identified FADCs.

Enzymes	Different key amino acid residues				
$OleT_{JE}$	46F	176L	294F	317A	402I
$OleT_{JH}$	47F	177L	295F	318A	403I
$OleT_{SQ}$	48I	178I	296Y	319A	404L
$OleT_{SA}$	47T	177I	296F	319V	405L

Blue color indicates that the five binding site residues are highly conserved in the two high activity P450 decarboxylases $OleT_{JE}$ and $OleT_{JH}$. Red color indicates the residues that are different in the lower activity enzyme $OleT_{SA}$.
