# Supplementary material for: Biochemical characterization of three new α-olefin-producing P450 fatty acid decarboxylases with a halophilic property
Source: Biotechnol Biofuels. 2019 Apr 8;12:79. doi: 10.1186/s13068-019-1419-6 (PMC6452516; doi:10.1186/s13068-019-1419-6)
Supplement: Supplementary file 2 — Additional file 2. The codon-optimized gene sequences of OleTJH, OleTSQ and OleTSA and their corresponding amino acid sequences. [file 13068_2019_1419_MOESM2_ESM.pdf]

The codon-optimized gene sequence of OleT$_{JH}$. *Nde*I and *Xho*I restriction sites are underlined. Start and stop codons are shown in red.

CATATGATGGCGAGCCTGAAACGCGATAAAGGCCTGGATAACACCCTGAAAGTGATG
AAACAGGGCTATCTGTATACCACCAACCAGCGCAACCGCTTAAACACCACCGTGTTT
GAAACCAAAGCGCTGGGCGGCAAACCTTTTGCAGTTGTGACCGGTAAAGAAGGCGCG
GAAATGTTTTATAACAACGACATTGTGCAGCGCGAAGGCATGCTGCCGAAACGCATT
GTGAACACCCTGTTTGGCAAAGGCGCGATTCATACCATTGATGGCAAAAAACACGTG
GATCGCAAAGCGCTGTTTATGAGCCTGATGACCGAAGGCAACCTGAACTATGTGCGC
GAACTGACCCGCACCTTATGGCAAGCAAACACCCAGCGCATGGAAAGCATGGATGAG
GTGAACATTTACCGCGAAAGCATTGTGCTGCTGACCAAAGTTGGTACTCGCTGGGCG
GGTGTTCAAGCACCTCCTGAACAGATTGAACGCATTGCGACCGATATGGACATTATG
ATCGACAGCTTTAAAGGCCTGGGCGGCGTGTTTAAAGGCTATAAGGAAAGCAAAGCG
GCGCGCCGTCGTGTTGAAGATTGGCTGGAAGATCAGATTCTGGAAACCCGCAAAGGC
AACATTCATCCGCCTGAAGGCACCGCGCTGTATGAATTTGCGCACTGGGAAGATTATC
TGGGCAACCCGATGGATTCACGCAACTGCGCGATTGATCTGATGAACACCTTTCGCCC
GCTGATTGCGATTAACCGCTTTGTGAGCTTTGGCCTGCTGGCGATGCATGATAATCCG
GTGAGCCGCGAAAAAATTAAAAGCGAGCCGGATTACGCGTATAAATTTGCGCAGGAA
GTGCGCCGCTATTATCCGTTTGTGCCGTTTTTACCGGGCAAAGCGAAAACCGATATTG
ATTTTCAGGGCGTGACCATTCCTGCGGGTCATGGCTTAGCGATTGATGTGTATGGCAC
CCTGCATGATGAAAGCCTGTGGGAAGATCCGAACGAATTTCGCCCGGAACGCTTTGA
AGGTTGGGATGGCAGCCCGTTTGATCTGATTCCTCAGGGCGGCGGCGATTATTGGACC
AACCATCGCTGTGCGGGCGAATGGATTACCGTGATTATCATGGAGGAAACCATGAAA
TATTTCGCGGAGAAGGTGACCTATGATGTGCCGGAACAGGATCTGACCGTGGATCTG
AACAGCATTCCGGGCTATGTGAAAAGCGGCTTCGTGATTAAAAACGTGCAGGAAGTG
GTGGATCGCCGCTAACTCGAG

The codon-optimized gene sequence of OleT$_{SQ}$. *Nde*I and *Xho*I restriction sites are underlined. Start and stop codons are shown in red.

CATATGGCGACCATTAAACGCGATAAAGGCCTGGATAACAGCGTGAAAGTGATGAAAC
AGGGCTATCTGTATACCACCAACCAGCGCGAACGCTTAGGTGTGACCGATGGCGTTTTT
GAAACCCGTGCGCTGGGCGGTAAACGCATTATTGTGCTGAGCGGCAAAGATGGCGCG
GAACTGTTTTATGATAACGACAAGATTGAACGCAGCGGCACCTTACCTAAACGTGTGG
TGAACACCCTGTTTGGCAAAGGCGCGATTCATACCACCACCGGCAAAGTGCATATTGA
TCGCAAAGCGCTGTTTATGAGCCTGATGACCGAAGGCAACCTGAAATATCTGCGCGAA
CTGACCCGCAACCTGTGGTTTGCAAACACCCAGCGCATGGAAAGCATGGATCAGGTG
AACGTGTATCGCGAAAGCATTATTGTGCTGACCAAAGTGGGTACTCGTTGGGCAGGCG
TTCAAGCGCCGGAAAAAGAAATTGAAAACATCGCGACCGACATGGATATTATGATCGA
CAGCTTCAAAGCGATTGGCAGCGCGTTTAAAGGCTATCGCGAAGCGAAAGCGGCGCG
TCGCCGTGTTGAAGATTGGCTGGAGGATCAGATTATTCAGACCCGCAAGGGCAAAATT
CATCCGCCGAAAGGCACCGCGCTGTATGAATTTGCGCACTGGAAAGATTATAAAGGCG
AACCGATGGATAGCCGCCTGTGCGGCATTGATCTGATGAACACCTTTCGCCCGCTGATT
GCGATTAACCGCTTTGTGGCGTTTGGCGCGCTGGCGATGTATGAAAATCCGGTGGCGC
GCGAAAAAATTAAACAGGATGACGACTACGCGTATATGTTTGCGCAGGAAGTGCGCCG
CTTTTATCCGTTTGTGCCGTATTTACCGGCGAAAGCGAAAGTGGACTTTCAGTACAAAG

GCTACGAGATTGAGAAAGACACCATGCTGGCGCTGGATATTTATGGCACCATGCATGAT
CCGAACGTGTGGGAAGATCCGAACGAATTTTATCCGGAGCGCTTCAAAGATTGGGATG
GCAGCCCGTTTGATTTAATTCCGCAGGGCGGCGGCGATTATCATACCAACCATCGCTGT
GCGGGCGAATGGATGACCGTGATTATTATGGAAGAAACCATGAAATATTTTGCGAGCCG
CATTAACTATGATGTGCCGGAACAGGATCTGACCGTGGATCTGAATAGCCTGCCGGGCT
ATATTAAAAGCGGCTTCGTGATTGAAAACGTGCAGGAAAACGTGGATCGCACC**TAA**CT
CGAG

The codon-optimized gene sequence of OleT$_{SA}$. *Nde*I and *Xho*I restriction sites are underlined. Start and stop codons are shown in red.

CAT**ATG**GGCAAACAGATTCCGAAAGATCGCGGCTTAGATAGCACCCTGAAAGTGCTGA
AAGAGGGCTATAAATATGTGCCGAACCGCCTGGAAAAATTTGACACCAACATCTTTGA
ACTGCGCGCGTTAGGTGGTCGTCGTACCGTTGTGTTTAGCGGCAAAGAAGCGGCGGAA
ATCTTTTATAACAACGAGCTGATTGAACGCCAGGGCACCTTACCTAAACGCGTGGTGA
ACACCCTGTTTGGCAAAGGCGCGATTCATACCACCGGCGGCAAAAAACATATTGATCG
CAAGGCGCTGTTTATGAGCCTGATGACCGAAGAAAACCTGGAATATCTGCGCGAACTG
ACCCGTAGCACCTGGTTTATGAACACCGAACGCATGGAACGCATGGATGAAGTGAACG
TGTACAAAGAGAGCATTATCCTGCTGACCAAAGTGGGTTTTCGCTGGGCGGGTATTATT
GCGAGCCCGGAAGAAATTGAAAGCTGCGCGAAAGATATGGATACCATGATCGACAGCT
TCAAAAACATTGGCACCGCGTTTAAAGGCTATCGCGAAGCGAAAAAAGCACGCGATC
GCGTGGAAACCTTTCTGGAGAACCAGATTATTGCGGTGCGCGAAGGTAAACTGACCCC
TCCTCAAGGTACCGCGCTGCATGAATTTAGCCACTGGGAAGATTTTGAAGGCAACCTG
ATGGATAGCCGCCTGTGCGCGATTGATCTGATGAACGTGGTGCGTCCGCTGGTTGCGAT
TAACCGCTTTGTGAGCTTTGGCGTGAAAGCGCTGCATGATTATCCGGGCGAAGCGGAA
AAAGTGTTTAACAACGAGAACGACTACGCGTATAAATTCGTGCAGGAAGTGCGCCGCT
TTTATCCGTTTGTGCCGTTTCTGCCGGGTAAAGCGGCGGTGGATATTGAATTTGACGGC
TACAAGATCGAAAAGGATACCTTCCTGGTGCTGGATATTTATGGCACCCTGCATCGTGA
AGGCCTGTGGGAAAACCCGGAACGCTTTTATCCGAACCGCTTTAGCGATTGGGATGGC
AGCCCGTTTGATTTAATTCCGCAGGGCGGCGGCGATTATTATACCAACCATCGCTGCGC
GGGTGAATGGATGACCATTATCATCATGGAGGAGAGCATGAAATATTTTGCGCGCAACA
TCAGCTATGACATGAAGAAGGATCAGGATCTGAGCGTGAACCTGAACAAACTGCCGG
GCCGTGTTGTTAGCGGCACCATTATTGAAAACGTGAACGCGCTGGTGAACCGCAATGT
GGAAAGCGTG**TAA**CTCGAG

Amino acid sequences of OleT$_{JH}$, OleT$_{SQ}$ and OleT$_{SA}$.

1) OleT$_{JH}$ (WP_092595307):

MMASLKRDKGLDNTLKVMKQGYLYTTNQRNRLNTTVFETKALGGKPFAVVTGKEGAE
MFYNNDIVQREGMLPKRIVNTLFGKGAIHTIDGKKHVDRKALFMSLMTEGNLNYVRELT
RTLWQANTQRMESMDEVNIYRESIVLLTKVGTRWAGVQAPPEQIERIATDMDIMIDSFKGL
GGVFKGYKESKAARRRVEDWLEDQILETRKGNIHPPEGTALYEFAHWEDYLGNPMDSRN
CAIDLMNTFRPLIAINRFVSFGLLAMHDNPVSREKIKSEPDYAYKFAQEVRRYYPFVPFLPG
KAKTDIDFQGVTIPAGHGLAIDVYGTLHDESLWEDPNEFRPERFEGWDGSPFDLIPQGGGD
YWTNHRCAGEWITVIIMEETMKYFAEKVTYDVPEQDLTVDLNSIPGYVKSGFVIKNVQEV
VDRR

2) OleT$_{SQ}$ (WP_092983663):
MATIKRDKGLDNSVKVMKQGYLYTTNQRERLGVTDGVFETRALGGKRIIVLSGKDGAEL
FYDNDKIERSGTLPKRVVNTLFGKGAIHTTTGKVHIDRKALFMSLMTEGNLKYLRELTRN
LWFANTQRMESMDQVNVYRESIIVLTKVGTRWAGVQAPEKEIENIATDMDIMIDSFKAIG
SAFKGYREAKAARRRVEDWLEDQIIQTRKGKIHPPKGTALYEFAHWKDYKGEPMDSRLC
GIDLMNTFRPLIAINRFVAFGALAMYENPVAREKIKQDDDYAYMFAQEVRRFYPFVPYLP
AKAKVDFQYKGYEIEKDTMLALDIYGTMHDPNVWEDPNEFYPERFKDWDGSPFDLIPQG
GGDYHTNHRCAGEWMTVIIMEETMKYFASRINYDVPEQDLTVDLNSLPGYIKSGFVIENV
QENVDRT

3) OleT$_{SA}$ (WP_049319149):
MGKQIPKDRGLDSTLKVLKEGYKYVPNRLEKFDTNIFELRALGGRRTVVFSGKEAAEIFY
NNELIERQGTLPKRVVNTLFGKGAIHTTGGKKHIDRKALFMSLMTEENLEYLRELTRSTW
FMNTERMERMDEVNVYKESIILLTKVGFRWAGIIASPEEIESCAKDMDTMIDSFKNIGTAF
KGYREAKKARDRVETFLENQIIAVREGKLTPPQGTALHEFSHWEDFEGNLMDSRLCAIDL
MNVVRPLVAINRFVSFGVKALHDYPGEAEKVFNNENDYAYKFVQEVRRFYPFVPFLPGK
AAVDIEFDGYKIEKDTFLVLDIYGTLHREGLWENPERFYPNRFSDWDGSPFDLIPQGGGD
YYTNHRCAGEWMTIIIMEESMKYFARNISYDMKKDQDLSVNLNKLPGRVVSGTIIENVN
ALVNRNVESV
